# Non-responders in a quitline evaluation are more likely to be smokers – a drop-out and long-term follow-up study of the Swedish National Tobacco Quitline

**DOI:** 10.1186/s12971-016-0070-2

**Published:** 2016-02-03

**Authors:** Eva Nohlert, John Öhrvik, Ásgeir R. Helgason

**Affiliations:** Centre for Clinical Research, Uppsala University, Västmanland County Hospital, 721 89 Västerås, Sweden; Department of Medicine, Karolinska Institutet, Stockholm, Sweden; Department of Public Health Sciences, Social Medicine, Karolinska Institutet and Centre for Epidemiology and Community Medicine, Stockholm County Council, Stockholm, Sweden; Reykjavik University, Reykjavik, Iceland

**Keywords:** Effectiveness, Intention-to-treat, Non-response, Per protocol, Proactive, Reactive, Responder-only analysis, Smoking, Telephone, Questionnaire

## Abstract

**Background:**

A previous randomized controlled trial (RCT) of the Swedish National Tobacco Quitline detected no significant differences in smoking cessation outcomes between proactive and reactive services at 12-month follow-up. However, the response rate was only 59 % and non-responders were over-represented in the proactive service. We performed a drop-out analysis to assess the smoking status of initial responders and non-responders.

**Methods:**

At 29–48 months after the first call, a postal questionnaire with six questions was sent to 150 random clients from the RCT database, with equal numbers from the proactive and reactive services as well as responders and non-responders at 12-month follow-up. Clients who did not return the questionnaire were contacted by telephone. The outcome measures were point prevalence (PP) and 6-month continuous abstinence (CA), and their associations with response status at 12 months were assessed by logistic regression.

**Results:**

The response rate was 74 % (111/150). Abstinence was significantly higher among initial responders than non-responders (PP 54 % vs. 32 %, *p* = .023 and CA 49 % vs. 21 %, *p* = .003). The odds ratios for initial responders vs. initial non-responders were, for PP = 2.5 (95 % CI 1.1–5.6, *p* = .024), and for CA = 3.7 (95 % CI 1.5–8.9, *p* = .004), after adjusting for proactive/reactive service.

**Conclusions:**

Non-responders to a 12-month follow-up smoking cessation questionnaire in a quitline setting were more likely to be smokers 1.5–3 years later. We propose a conservative correction factor of 0.8 for self-reported abstinence in telephone-based cessation studies if the response rate is approximately 55–65 %.

## Background

Tobacco control remains a critical public health challenge, and thus encouraging smoking cessation is crucial for reducing tobacco-related mortality and morbidity in future years [1]. In Sweden, the prevalence of adult daily smoking has steadily declined since the 1980s to 10 % in 2014 [2]. However, in Sweden, which has a population of nearly 10 million, 1.6 million people still use tobacco (cigarettes and/or snus [moist snuff]) every day and it is estimated that 12,000 people die from smoking-related diseases each year (33 per day) [3].

Telephone-based counselling (via quitlines) is an evidence-based option for tobacco cessation support, which is both effective and cost-effective [4, 5]. Quitlines usually offer both a reactive service where only incoming calls are attended and a proactive service that offers call-backs. According to meta-analyses, the odds of quitting are 40 % higher for smokers who call quitlines and who receive multiple proactive counselling compared with controls who receive brief counselling and/or self-help materials by mail [4, 5].

Postal questionnaires are commonly used to measure the effectiveness of quitlines. In Nordic countries, the response rates to questionnaires have generally decreased during recent decades, which might affect the trustworthiness of studies employed to evaluate effectiveness [6]. The North American Quitline Consortium (NAQC), an organization that provides leadership in promoting evidence-based quitline services for the United States and Canada, recommend 50 % as a minimum survey response rate for studies reporting quit rates [7]. In 2013, six out of 36 quitlines in the USA and Canada achieved the recommended 50 % response rate, with rates between 13 and 59 % and an average of 41 % [8].

In Sweden, ever since it was found that smokers are over-represented among non-responders to population-based questionnaire assessments of living conditions [9], the usual practice has been to treat non-responders as smokers in intention-to treat (ITT) analyses. This conservative approach will not overestimate the treatment effect but it may yield underestimates because not all non-responders are likely to be smokers. Indeed, a previous drop-out study of the Swedish National Tobacco Quitline (SNTQ) concluded that treating non-responders at 12-month follow-up as smokers might significantly underestimate the true effect of cessation treatment [10].

The SNTQ is a free service that operates nationwide, which is partly financed by the Stockholm County Council Health Service but the Swedish Government is the main financing body. The SNTQ started in 1998 with a reactive service*.* In 1999, a proactive service was introduced and clients could choose between a reactive or proactive service. Previous studies have reported a ca 30 % point prevalence abstinence (responders only) at 12-month follow-up, with a cost per life-year saved of about 400 USD [11, 12], and the proactive service was considered to be marginally more effective than the reactive service for women but not for men [11]. However, these results are based on non-randomized studies where the clients could choose the service that they wanted.

To facilitate a better comparison of the effectiveness of the higher-intensity proactive service and that of the lower-intensity reactive service, a randomized controlled trial was performed during 2009–2010, in the sequel called the RCT-study [13]. No statistically significant differences in smoking cessation outcomes were detected at the 12-month follow-up between the proactive and reactive services, in terms of point prevalence or continuous abstinence, or ITT or responder-only analyses.

However, there were some differences in the baseline characteristics of the responders and non-responders to the 12-month follow-up [13]. In particular, it was interesting that although there were no differences between the proactive and reactive services in terms of data collection and recruitment, the clients who received the proactive service were significantly less likely to respond to the 12-month follow-up questionnaire [13].

Overall, the proportion of responders to the 12-month follow-up postal questionnaires sent by the SNTQ has decreased over time from about 70 % in 1999 to 60 % at the time of the present study [11, 13, 14]. This decline in the response rate suggests the need for a new non-responder analysis to assess the possible effects on the proportion of clients who are still smokers among the non-responders. In addition, as non-responders were over-represented in the proactive service [13], a comparison between the different services is required.

The present study was the second non-responder/drop-out analysis to be conducted at the SNTQ. The main hypothesis tested in this study is that non-responders to the 12-month follow-up are more likely to be smokers at the time of the non-response analysis compared with responders to the 12-month follow-up questionnaire. We also assessed whether the two different treatment services (proactive and reactive) yielded different proportions of present-smokers among the non-responders.

## Methods

### Standard SNTQ process

The SNTQ and the counselling process have been described previously [11, 13], but we provide a brief description of the standard SNTQ process.

All calls to the SNTQ are registered in a computerized database. When a tobacco user calls to discuss his/her personal tobacco behaviour, the counsellor asks whether the client would like to sign up for cessation support. If the client gives verbal consent, their preference for call-back (proactive service) or no call-back (reactive service) is recorded, and a registration form, which includes the baseline questionnaire, is mailed to them. The return of the baseline questionnaire is regarded as informed consent and the client is included in a study base to assess effectiveness. Twelve months after the first call, a follow-up questionnaire is sent by mail to the client. Non-responders to the baseline or follow-up questionnaire receive up to two reminders, one by mail, and one by telephone.

### The RCT study

**Fig. 1:**
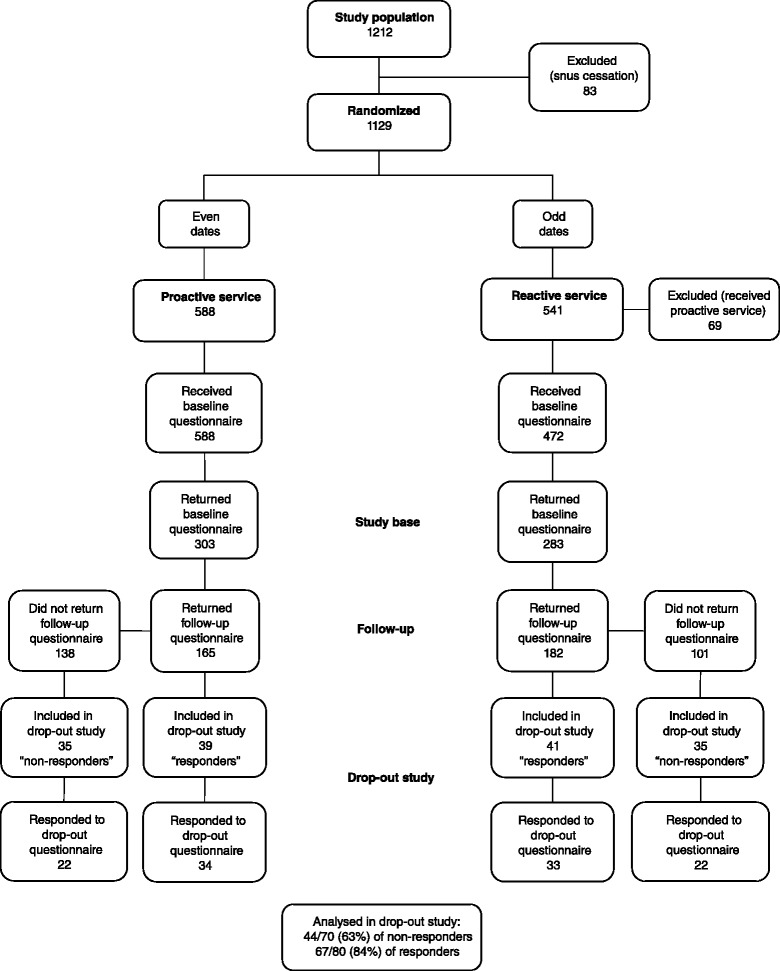
Flowchart illustrating the process followed in the RCT and the drop-out study. Clients were included from February 2009 to September 2010. The drop-out study was performed in February to March 2013

The initial RCT study was performed as part of the normal operation at the SNTQ, where the only difference was that callers were not offered a choice of callbacks or no callbacks. Instead, only those who called on even dates were offered callbacks, i.e., the proactive service, and those who called on odd dates were informed that they could call back themselves whenever they liked, i.e., the reactive service. The study base comprised the 586 clients who returned the baseline questionnaire during the recruitment period: 303 from the proactive service group and 283 from the reactive service group (Fig. 1) [13].

### The drop-out study

The drop-out study was performed between 2 years 5 months and 4 years (29–48 months) after the client’s first call, and thus it may be defined as both a drop-out study and a long-term follow-up of the SNTQ results. A questionnaire was compiled with six questions, all of which had been validated and used previously in the baseline and 12-month follow-up questionnaires in the initial RCT study [13]. The questions included smoking habits, use of snus and pharmaceuticals (nicotine replacement therapy (NRT), Champix® and Zyban®), and intention to quit (if still smoking). The questionnaire was pre-tested by six clients.

A random sample of 60 % of the clients in the RCT database was drawn from each of the four categories; proactive 12-month responders, proactive 12-month non-responders, reactive 12-month responders, and reactive 12-month non-responders. The national population register was then checked for deaths and emigration details. Since the clients were drawn randomly their order is random and thus we drew the clients consecutively from each of the four categories. The target was to get around 40 from each category. This was based on previous power calculations where we assumed at least 20 % difference between the groups.

The questionnaires were posted to the clients with a cover letter and the clients were asked to return the answered questionnaire in an enclosed stamped envelope. Those who did not return the questionnaire were contacted by telephone and they could choose whether to return the questionnaire or to answer the questions during a structured telephone interview. The six clients from the pre-test (two in the proactive group and four in the reactive group, where all six were initial responders) were also included because no changes to the questionnaire were required after the pre-test. The flow chart of the study is presented in Fig. 1.

### Abstinence questions and outcomes

Abstinence was assessed by two questions: 1) “Have you smoked (one or more deep drags) during the past 7 days?” with response alternatives of “no, not at all”, “yes, but not daily”, and “yes daily”; and 2) “When did you take your last puff?” with response alternatives of “0–7 days ago”, “more than 7 days but less than 6 months ago”, “6–12 months ago”, and “more than 12 months ago”. The outcome measures were point prevalence abstinence (not a puff in the past 7 days) and 6-month continuous abstinence (not a puff in the past 6 months) at the 12-month follow-up.

The study was approved by the Ethical Committee at the Karolinska Institutet (Dnr 00-367).

### Data analysis

SPSS (version 22) was used to conduct all of the statistical analyses and significance differences were accepted at *p* < .05 (two-sided). In the comparisons of responders and non-responders, the Mann–Whitney *U*-test was used for age and the Chi-Square test was used for categorical variables, although Fisher’s exact test was applied for the use of snus. The association between outcome and response status at 12 months after adjusting for treatment was assessed by logistic regression. McNemar’s test was used to compare the outcomes (point prevalence and 6-month continuous abstinence) in those who responded to both the 12-month follow-up in the RCT study and to the drop-out study.

## Results

The response rate was 74 % (111/150). Sixty-two clients returned the questionnaires without reminders and 49 responded to the questionnaire after a telephone reminder. Significantly more initial responders than initial non-responders in the proactive service replied to the drop-out study (87 vs. 63 %, *p* = .015). The difference was similar for the reactive service but it did not reach significance (80 vs. 63 %, *p* = .087). The response pattern in the drop-out study is presented in Table 1.

**Table 1:** Response patterns in the drop-out study

% (n/N)	Total			Proactive			Reactive		
	Resp^a^ *n* = 80	Non-resp^b^ *n* = 70	*p* ^c^	Resp^a^ *n* = 39	Non-resp^b^ *n* = 35	*p* ^c^	Resp^a^ *n* = 41	Non-resp^b^ *n* = 35	*p* ^c^
Total number of replies to the drop-out study	84 (67/80)	63 (44/70)	.004	87 (34/39)	63 (22/35)	.015	80 (33/41)	63 (22/35)	.087
- replies without telephone reminder	64 (43/67)	43 (19/44)	.029	59 (20/34)	45 (10/22)	.327	70 (23/33)	41 (9/22)	.034
- replies after telephone reminder	36 (24/67)	57 (25/44)		41 (14/34)	55 (12/22)		30 (10/33)	59 (13/22)	
Proportion of those who got a telephone reminder who replied	69 (24/35)	53 (25/47)	.160	74 (14/19)	52 (12/23)	.153	63 (10/16)	54 (13/24)	.601

^a^Resp: responders (both baseline and 12 months)

^b^Non-resp: non-responders (baseline but not 12 months)

^c^Differences between responders and non-responders were tested with the chi-square test

Almost half (45 %) of those who responded to the drop-out study were smoke-free 29–48 months after their first call. Abstinence was significantly higher among responders than among non-responders to the 12-month follow-up in the original RCT study. Point prevalence was 54 % among initial responders and 32 % among initial non-responders (*p* = .023), and 6-month continuous abstinence was 49 % among initial responders and 21 % among initial non-responders (*p* = .003). There were no differences in pharmaceutical or snus use between the groups. Intention to quit among those still smoking was equal in both groups (Table 2).

**Table 2:** Abstinence and other pertinent results obtained from the drop-out study

% (n/N)	Total	Responders (both baseline and 12 months)	Non-responders (baseline but not 12 months)	*p*-value^*^
Point prevalence	45 (50/111)	54 (36/67)	32 (14/44)	.023
- in proactive service	41 (23/56)	50 (17/34)	27 (6/22)	.091
- in reactive service	49 (27/55)	58 (19/33)	36 (8/22)	.123
6-month continuous abstinence	38 (42/110)	49 (33/67)	21 (9/43)	.003
- in proactive service	34 (19/56)	44 (15/34)	18 (4/22)	.045
- in reactive service	43 (23/54)	55 (18/33)	24 (5/21)	.026
Women	75 (83/111)	72 (48/67)	79 (35/44)	.348
Age (median; q_1_-q_3)_	50; 38–58	51; 41–59	45; 34–55	.044
Pharmaceutical^a^ use in the past 7 days	20 (22/110)	21 (14/67)	19 (8/43)	.769
Pharmaceutical^a^ use since first call to SNTQ:				
- no	41 (45/109)	42 (28/66)	39 (17/43)	.925
- yes, < 5 weeks	19 (21/109)	18 (12/66)	21 (9/43)	
- yes, ≥ 5 weeks	39 (43/109)	39 (26/66)	39 (17/43)	
Snus use in the past 7 days	11 (12/109)	12 (8/66)	9 (4/43)	.761
Intention to quit (among still smokers):				
- action + preparation	49 (30/61)	53 (17/32)	45 (13/29)	.517
- contempl. + precont ^b^	51 (31/61)	47 (15/32)	55 (16/29)	

*N* = 150, response 111 (74 %). First call February 2 2009 to September 23 2010. Follow-up February to March 2013 (⇒2 years 5 months to 4 years)

^*^Differences between responders and non-responders were tested with the chi-square test for all variables except age which was tested with the Mann–Whitney *U*-test and snus use which was tested with Fisher’s exact test

^a^NRT, Zyban®, Champix®

^b^Contempl.: contemplation. Precont.: precontemplation

According to the regression analysis, responders to the initial 12-month follow-up were more than twice as likely to be point prevalence abstinent compared with non-responders (odds ratio (OR) 2.5, 95 % confidence interval (CI) 1.1–5.6, *p* = .024) when adjusted for proactive/reactive service. The adjusted odds ratio was even higher for 6-month continuous abstinence (OR 3.7, 95 % CI 1.5–8.9, *p* = .004) (Table 3).

**Table 3:** Logistic regression analysis of abstinence in the drop-out study (OR: odds ratio; CI: confidence interval)

	Point prevalence		6-month continuous abstinence	
	OR (95 % CI)	*p*-value	OR (95 % CI)	*p*-value
Proactive vs. reactive (ref) service	0.7 (0.3–1.5)	.396	0.7 (0.3–1.5)	.351
Responders vs. non-responders (ref)	2.5 (1.1–5.5)	.025	3.7 (1.5–8.8)	.004
Responders vs. non-responders (ref) after adjusting for service	2.5 (1.1–5.6)	.024	3.7 (1.5–8.9)	.004

Fully 70 % of those who were smoke-free at the initial 12-month follow-up were still smoke-free after 29–48 months. In addition, approximately one third of those who were smoking at 12-month follow-up reported to be smoke-free in the present study (Table 4). The pattern was almost the same for the proactive and reactive services. The abstinence at the 12-month follow-up in the original RCT study and a comparison of the baseline characteristics among responders and non-responders to the 12-month follow-up is previously reported [13].

**Table 4:** Transition of point prevalence abstinence and 6-month continuous abstinence among responders at both the 12-month follow-up and in drop-out study

% (n/N)	Point prevalence abstinence		6-month continuous abstinence	
	Smoke-free in drop-out study	Smoker in drop-out study	Smoke-free in drop-out study	Smoker in drop-out study
Smoke-free at 12-month follow-up	**72** (23/32)	**28** (9/32)	**74** (20/27)	**26** (7/27)
Smoker at 12-month follow-up	**37** (13/35)	**63** (22/35)	**33** (13/40)	**67** (27/40)
	McNemar’s Test *p* = .523		McNemar’s Test *p* = .263	

The observed power in the present study was 78 % for point prevalence abstinence and 96 % for 6-month continuous abstinence (α = 5 %) (Table 2).

## Discussion

A clear response bias was detected in the present drop-out study, where the initial responders were significantly more likely to be smoke-free than the initial non-responders, a difference of approximately 20 %. However, almost one third of the initial non-responders who participated in the present study reported to be smoke-free at the time of the drop-out assessment (Table 2).

A responder at the 12-month follow-up was 2.5 (in terms of point prevalence) and 3.7 (in terms of 6-month continuous abstinence) times more likely to be abstinent than a non-responder in the drop-out study, after adjusting for proactive/reactive service (Table 3).

The stability of the long-term abstinence rate was notable in this long-term follow-up. Among responders at the 12-month follow-up in the original RCT study, 47 % were smoke-free [13]. Among responders to the present drop-out study, 45 % were smoke-free at 29–48 months after their first call to the SNTQ (Table 2).

A previous drop-out study at the SNTQ found only a small difference (31 % vs. 28 %) in favour of the responders [10]. The observed difference between the results of these two studies in terms of the reported quit rates at the time of drop-out analysis may be explained as follows. In the previous study, the initial response rate at 12-month was higher, i.e., 71 % compared with 59 % in the present study. In addition, the time between the 12-month follow-up and the drop-out analysis was considerably shorter in the previous study, i.e., 4 months compared with 29–48 months in the present study. Otherwise, the data collection methods were similar and the definition of the study base and the questions used to assess abstinence were the same in both studies.

As shown in Table 4, a large proportion of non-smokers at 12 months remained abstinent (approximately seven out of ten), and one out of three smokers at 12 months reported that they were non-smokers in the current long-term follow-up. The stable long-term abstinence rate from 12-month follow-up until the drop-out study agreed with our previous long-term (5–8 years) follow-up of smokers treated at dental clinics, where we detected a stable and somewhat increasing proportion of quitters after the initial 12-month follow-up (with 8 % for 5 years) [15].

Among the initial responders to the 12-month follow-up, the abstinence rate was considerably higher at the drop-out assessment compared with that at the 12-month follow-up in the original RCT study (point prevalence 54 % vs. 47 %, 6-month continuous abstinence 49 % vs. 35 %) (Table 2 and [13]). This may be explained by positive selection; that the responders in both assessments were those who were most likely to be abstinent. An increase in proportion of abstinent smokers over time, as noted in previous long-term follow-ups [15–17], may also partly explain the difference.

In ITT-analyses that treated all of the non-responders in the drop-out analysis as smokers, the point prevalence abstinence was 33 % (50/150) (initial responders 45 % vs. initial non-responders 20 %, *p* = .001) and the 6-month continuous abstinence was 28 % (42/149) (initial responders 41 % vs. initial non-responders 13 %, *p* < .001) (not shown in the tables). Thus, the ITT abstinence rates were approximately 10 % lower than the abstinence rates among the responders in the present study (Table 2). Based on a comparison of 111 clients who responded to the drop-out study and 39 who did not, we found that responders were significantly older and more likely to be smoke-free at 12 months. However, we found no significant differences in terms of the sex distribution, baseline use of cigarettes, snus or pharmaceuticals, and exposure to second-hand smoke (not shown in the tables).

ITT analyses in tobacco cessation studies will not overestimate effectiveness; but the true or real abstinence rates are probably between the results of ITT analyses and per protocol-/responder-only analyses. Therefore, it may be possible to estimate the true/real abstinence rate by correcting for the responder-only abstinence. Thus, we propose the use of a correction factor, which can be obtained by calculating the relationship between abstinence in the total drop-out sample and among the initial responders. The point prevalence abstinence was 45 % in the total drop-out sample and 54 % among initial responders, so we propose that 45/54 = 0.83 can be used as a correction factor. The 6-month continuous abstinence was 38 % in the total drop-out sample and 49 % among initial responders, i.e., 38/49 = 0.78 (Table 2). Assuming that the relationship between the prevalence of responders and non-responders is the same at 12 months and at long-term (drop-out) follow-up, we propose a conservative correction factor of *0.8* for self-reported abstinence in telephone-based cessation studies with a response rate of approximately 55–65 %.

In the present study, we also aimed to assess whether the two different support protocols (proactive vs. reactive service) yielded different proportions of current smokers among the non-responders. However, there was no evidence of that, since the logistic regression analysis showed that response status was the main variable for abstinence after adjusting for service type (Table 3).

The decrease in the response rate at the SNTQ over time is a matter of concern and has appeared although follow-up procedures have not changed. Even though the response rate is relatively normal in studies like this [18–20], there is a possibility of bias due to differential loss to follow-up because of differences between responders and non-responders [13]. The responders to the 12-month follow-up were significantly older, more likely to be smoke-free at the first call, pharmaceutical users, and not exposed to second-hand smoke. The responders also smoked fewer cigarettes/day but they had been smokers for a longer period of time.

In the present drop-out study, two thirds of the initial responders answered without a telephone reminder, whereas almost two thirds of initial non-responders answered after a telephone reminder. The only difference between those who responded before and after the telephone reminder was that the early responders were more likely to be smoke-free at 12 months.

A telephone reminder resulted in answers from 69 % of the responders and 53 % of the non-responders. The response rate increased from 41 % (62/150) before the telephone reminder to 74 % (111/150) after the reminder (Table 1). This increase is comparable to health surveys with multiple reminders [21–23]. Systematic reviews have shown that reminder letters and telephone contact, personalized letters, short questionnaires, and stamped return envelopes increase the response rate to postal questionnaires [24–26].

The abstinence rates were higher, but not significantly, among those who answered without a telephone reminder than among those who answered after a reminder (point prevalence 52 % (32/62) vs. 37 % (18/49), *p* = .118 and 6-month continuous abstinence 46 % (28/61) vs. 29 % (14/49), *p* = .063) (not shown in the tables). In our previous long-term follow-up study of smokers treated at dental clinics, the abstinence rates were also significantly higher among questionnaire responders than subsequent telephone responders (*p* = .001) [15], but this could also indicate a difference between early and late responders.

A quite recent study from three American quitlines (Minnesota, Hawaii, and Florida) reports higher, but not statistically significant quit rates among earlier than later responders [20]. The participants were offered incentives and abstinence measure was 30-day point prevalence after 7 months (figures between 20 and 40 %), making a comparison with our quit rates rather meaningless.

A strength of the present study is the relatively high response rate, but it was not 100 %, so it is also a limitation because of the possible bias due to differential losses to follow-up. Another strength is that the present study included a long-term follow-up of the quitline results, which is very rare to the best of our knowledge. A limitation is that abstinence was self-reported, but self-reporting is considered to be accurate in most smoking cessation studies and biochemical verification might not be desirable in studies where mail, telephone, or the Internet are the optimal data collection methods [27]. In addition, the outcome measure was smoking cessation, so the use of other tobacco products (e.g., snus) and alternative nicotine-delivery methods (e.g., NRT or electronic “cigarette” vapour) would have complicated any cotinine measurements performed to validate the self-reported data. A further limitation is that the response rate in the drop-out study was higher among the initial responders (84 %) than that among the initial non-responders (63 %).

## Conclusions

Non-responders to a 12-month follow-up smoking cessation questionnaire in a quitline setting were more likely to be smokers 1.5–3 years later. We propose a conservative correction factor of 0.8 for self-reported abstinence in telephone-based cessation studies if the response rate is approximately 55–65 %.

## References

[1] World Health Organization (2011). WHO report on the global tobacco epidemic.

[2] Swedish National Public Health Survey - Health on Equal Terms, 2014 [Internet]. Folkhälsomyndigheten [Public Health Agency of Sweden]. 2015 [cited January 22, 2015]. Available from: http://www.folkhalsomyndigheten.se/amnesomraden/statistik-och-undersokningar/enkater-och-undersokningar/nationella-folkhalsoenkaten/levnadsvanor/tobaksvanor/.

[3] The National Board of Health and Welfare (2014). Register data of the harmful effects of tobacco smoking.

[4] Fiore MC, Jaén CR, Baker TB, Bailey WC, Benowitz NL, Curry SJ (2008). Treating tobacco use and dependence: 2008 update. Clinical practice guideline.

[5] Stead LF, Hartmann-Boyce J, Perera R, Lancaster T (2013). Telephone counselling for smoking cessation. Cochrane Database Syst Rev.

[6] Sogaard AJ, Selmer R, Bjertness E, Thelle D (2004). The Oslo Health Study: The impact of self-selection in a large, population-based survey. Int J Equity Health.

[7] NAQC (2009). Measuring quit rates. Quality Improvement Initiative.

[8] NAQC. Results from the 2013 NAQC Annual Survey of Quitlines: North American Quitline Consortium (NAQC). 2014. [January 19, 2016]. Available from: http://www.naquitline.org/?page=2013Survey

[9] Tillgren P, Haglund BJ, Lundberg M, Romelsjo A (1996). The sociodemographic pattern of tobacco cessation in the 1980s: results from a panel study of living condition surveys in Sweden. J Epidemiol Community Health.

[10] Tomson T, Bjornstrom C, Gilljam H, Helgason A (2005). Are non-responders in a quitline evaluation more likely to be smokers?. BMC Public Health.

[11] Helgason AR, Tomson T, Lund KE, Galanti R, Ahnve S, Gilljam H (2004). Factors related to abstinence in a telephone helpline for smoking cessation. Eur J Public Health.

[12] Tomson T, Helgason AR, Gilljam H (2004). Quitline in smoking cessation: a cost-effectiveness analysis. Int J Technol Assess Health Care.

[13] Nohlert E, Ohrvik J, Helgason AR (2014). Effectiveness of proactive and reactive services at the Swedish National Tobacco Quitline in a randomized trial. Tob Induc Dis.

[14] Lindqvist H, Forsberg LG, Forsberg L, Rosendahl I, Enebrink P, Helgason AR (2013). Motivational interviewing in an ordinary clinical setting: A controlled clinical trial at the Swedish National Tobacco Quitline. Addict Behav.

[15] Nohlert E, Ohrvik J, Tegelberg A, Tillgren P, Helgason AR (2013). Long-term follow-up of a high - and a low-intensity smoking cessation intervention in a dental setting--a randomized trial. BMC Public Health.

[16] Murray RP, Connett JE, Rand CS, Pan W, Anthonisen NR (2002). Persistence of the effect of the Lung Health Study (LHS) smoking intervention over eleven years. Prev Med.

[17] Miguez MC, Becona E (2008). Abstinence from smoking ten years after participation in a randomized controlled trial of a self-help program. Addict Behav.

[18] Willemsen MC, van der Meer RM, Bor S (2008). Description, effectiveness, and client satisfaction of 9 European Quitlines: Results of the European Smoking Cessation Helplines Evaluation Project (ESCHER).

[19] Ferguson J, Docherty G, Bauld L, Lewis S, Lorgelly P, Boyd KA (2012). Effect of offering different levels of support and free nicotine replacement therapy via an English national telephone quitline: randomised controlled trial. BMJ.

[20] Lien RK, Schillo BA, Goto CJ, Porter L (2016). The effect of survey nonresponse on quitline abstinence rates: Implications for practice. Nicotine Tob Res.

[21] Brogger J, Bakke P, Eide GE, Gulsvik A (2003). Contribution of follow-up of nonresponders to prevalence and risk estimates: a Norwegian respiratory health survey. Am J Epidemiol.

[22] Christensen AI, Ekholm O, Kristensen PL, Larsen FB, Vinding AL, Glumer C (2015). The effect of multiple reminders on response patterns in a Danish health survey. Eur J Public Health.

[23] Verlato G, Melotti R, Olivieri M, Corsico A, Bugiani M, Accordini S (2010). Asthmatics and ex-smokers respond early, heavy smokers respond late to mailed surveys in Italy. Respir Med.

[24] Edwards P, Roberts I, Clarke M, DiGuiseppi C, Pratap S, Wentz R (2002). Increasing response rates to postal questionnaires: systematic review. BMJ.

[25] Nakash RA, Hutton JL, Jorstad-Stein EC, Gates S, Lamb SE (2006). Maximising response to postal questionnaires - a systematic review of randomised trials in health research. BMC Med Res Methodol.

[26] Edwards PJ, Roberts I, Clarke MJ, Diguiseppi C, Wentz R, Kwan I (2009). Methods to increase response to postal and electronic questionnaires. Cochrane Database Syst Rev.

[27] Society for Research on Nicotine and Tobacco Subcommittee on Biochemical Verification (2002). Biochemical verification of tobacco use and cessation. Nicotine Tob Res.

